# Fission Yeast CENP-C (Cnp3) Plays a Role in Restricting the Site of CENP-A Accumulation

**DOI:** 10.1534/g3.118.200486

**Published:** 2018-06-20

**Authors:** Michiko Suma, Teppei Kitagawa, Yukiko Nakase, Norihiko Nakazawa, Mitsuhiro Yanagida, Tomohiro Matsumoto

**Affiliations:** * Radiation Biology Center, Graduate School of Biostudies, Kyoto University, Yoshida-Konoe cho, Sakyo ku, Kyoto, Japan, 606-8501; † G0 cell unit, Okinawa Institute of Science and Technology Graduate University, Onna-son, Okinawa, Japan, 904-0495

**Keywords:** centromere, CENP-A, CENP-C, and fission yeast

## Abstract

The centromere is a chromosomal locus where a microtubule attachment site, termed kinetochore, is assembled in mitosis. In most eukaryotes, with the exception of holocentric species, each chromosome contains a single distinct centromere. A chromosome with an additional centromere undergoes successive rounds of anaphase bridge formation and breakage, or triggers a cell cycle arrest imposed by DNA damage and replication checkpoints. We report here a study in *Schizosaccharomyces pombe* to characterize a mutant (*cnp3-1*) in a gene encoding a homolog of mammalian centromere-specific protein, CENP-C. At the restrictive temperature 36°, the Cnp3-1 mutant protein loses its localization at the centromere. In the *cnp3-1* mutant, the level of the Cnp1 (a homolog of a centromere-specific histone CENP-A) also decreases at the centromere. Interestingly, the *cnp3-1* mutant is prone to promiscuous accumulation of Cnp1 at non-centromeric regions, when Cnp1 is present in excess. Unlike the wild type protein, Cnp3-1 mutant protein is found at the sites of promiscuous accumulation of Cnp1, suggesting that Cnp3-1 may stabilize or promote accumulation of Cnp1 at non-centromeric regions. From these results, we infer the role of Cnp3 in restricting the site of accumulation of Cnp1 and thus to prevent formation of *de novo* centromeres.

The centromere is a chromosomal locus on which a microtubule attachment site, termed kinetochore, is assembled in mitosis. It is therefore an essential component for faithful segregation of chromosomes. A variant of histone H3, CENP-A, is specifically incorporated into centromeric chromatin and plays an important role as the base of the kinetochore assembly. (Black and Cleveland 2011; Quénet and Dalal 2012; Catania and Allshire 2014). Distribution of CENP-A must be strictly regulated so that the centromere is positioned and organized appropriately for each species. Although it is generally accepted that centromeres are defined by a sequence-independent epigenetic manner (Allshire and Karpen 2008; Black and Cleveland 2011; Perpelescu and Fukagawa 2011), the underlying mechanisms to deposit and maintain CENP-A at the right place are still elusive.

In most eukaryotes, with the exception of holocentric species, each chromosome contains a single distinct centromere. It has been reported that a chromosome with an extra centromere (dicentric chromosome) causes deleterious problems such as chromosome breakage (Thrower and Bloom 2001; Thrower *et al.* 2003; Pobiega and Marcand 2010) and cell cycle arrest (Sato *et al.* 2012). A dicentric chromosome can also be generated by translocation of a centromere followed by meiotic recombination. To avoid these problems, each chromosome contains a single centromere whose position is maintained through generations. As CENP-A histone is a major component found in centromeric chromatin, distribution of CENP-A fundamentally influences formation and positioning of the centromere.

Recent studies have shown that CENP-A accumulation can be found in non-cetromeric chromatin as well. In chicken DT-40 cells, minor accumulation of CENP-A is detected around the native centromeres. When the native centromere is experimentally removed, a neocentromere is occasionally assembled by using the minor accumulation of CENP-A as a seed (Shang *et al.* 2013). In budding yeast, Cse4, a homolog of mammalian CENP-A, is enriched at promoters that contain histone H2A (Hildebrand and Biggins 2016). Psh1 is an E3 ubiquitin ligase (Hewawasam *et al.* 2010 ; Ranjitkar *et al.* 2010) that blocks stable Cse4 incorporation into these promoters by targeting mislocalized Cse4 for degradation (Collins *et al.* 2004). The ubiquitin system is a conserved pathway for preventing ectopic CENP-A accumulation in other organisms including fission yeast and fly (Gonzalez *et al.* 2014; Moreno-Moreno *et al.* 2006).

In fission yeast, overexpression of CENP-A causes the promiscuous incorporation of CENP-A near heterochromatic regions (Choi *et al.* 2012; Castillo *et al.* 2013; Gonzalez *et al.* 2014). Most of these loci with CENP-A accumulation do not transform into a neocentromere. It has been shown that the histone H2A variant (Pht1), a NAP family protein (Ccp1) and a histone chaperone FACT play a role in antagonizing CENP-A loading at non-centromeric regions (Choi *et al.* 2012; Ogiyama *et al.* 2013; Dong *et al.* 2016). These factors thus likely prevent formation of neocentromeres.

Interestingly, sub-telomeric regions in fission yeast can accommodate neocetromeres when the native centromere is disrupted (Ishii *et al.* 2008). Subsequent study has revealed that stable association of CENP-A chaperon, Scm3, facilitates neocetromere formation (Ogiyama *et al.* 2013). Nonetheless, neocentromere formation is a very rare event (several in ten thousand cells in fission yeast), suggesting that neocetromere formation is strongly suppressed likely by multiple mechanisms.

Having been interested in mechanisms to maintain appropriate distribution of CENP-A and prevent formation of *de novo* centromeres, we have taken a genetic approach in fission yeast model system. We assumed that a mutant unable to maintain proper distribution of CENP-A would be sensitive to expression of CENP-A at a high level. A strain ectopically expressing fission yeast CENP-A (Cnp1) tagged with GFP (GFP-Cnp1) from an inducible promoter, nmt1, was mutagenized and survivors were screened for mutants, which became temperature sensitive upon overexpression of GFP-Cnp1. We have previously reported a mutant (*rpt3-1*) identified through the screen (Kitagawa *et al.* 2014). Unlike the wild type fission yeast in which the localization of CENP-A is limited to the central region spanning 10–20 kb of the centromere, the *rpt3-1* mutation allows spread of CENP-A localization at the centromere (40–70 kb). Likely due to abnormal distribution of CENP-A, the *rpt3-1* mutant exhibits chromosome instability and enhanced gene silencing. In this study, we have characterized another mutant identified through the same screen. Our analyses have shown that the mutation in the *cnp3^+^* gene encoding a fission yeast homolog of the mammalian CENP-C allows accumulation of Cnp1 at non-centromeric regions. We thus propose a role of the wild type Cnp3 in restricting the site of accumulation of Cnp1 to prevent formation of *de novo* centromeres.

## Materials and Methods

### Strain, culture media and strain construction

An *S. pombe* haploid wild-type strain *SP6* (*h- leu1-32*) and its derivatives used in this study are listed in Table 1. The culture media were complete YES, minimal EMM2, and sporulation medium MEA and SPA (Moreno *et al.* 1991).Transformation was done using the lithium method (Ito *et al.* 1983). The cell number was measured using a hematology analyzer (Sysmex F-520P and Beckman multisizer 3 coulter counter).

**Table 1:** *S. pombe* strains used in this study

Strain		Genotype	Source and reference
SP6	*h^-^*	*leu1-32*	Laboratory stock
MS01	*h^-^*	*leu1-32* transformed with *pREP41-GFP-H3.3*	This work
MS02	*h^-^*	*leu1-32* transformed with *pREP41-GFP-cnp1*	This work
MS03	*h^-^*	*leu1-32 cnp3-1* transformed with *pREP41-H3.3*	This work
MS04	*h^-^*	*leu1-32 cnp3-1* transformed with *pREP41-cnp3-1*	This work
MS168	*h^+^*	*leu1-32*::*nmtGFP-cnp1-leu1^+^ cnp3*::*kanR ade6-M216*	This work
MS213	*h^-^*	*leu1-32 lys*::*nmtGFP-cnp1-lys1^+^ cnp3-mCherry-leu1^+^*	This work
MS216	*h^+^*	*leu1-32*::*nmtGFP-cnp1-leu1^+^ ade6-M216*	This work
MS217	*h^-^*	*leu1-32 lys*::*nmtGFP-cnp1-lys1^+^ cnp3-1-mCherry-leu1^+^*	This work
MS262	*h^+^*	*leu1-32*::*nmtGFP-cnp1-leu1^+^ cnp3-1 ade6-M216*	This work
MS343	*h^-^*	*leu1-32 GFP-cnp1-hph*	Laboratory stock
MS344	*h^-^*	*leu1-32 GFP-cnp1-hph cnp3-1*	This work
MS413	*h^-^*	*leu1-32 cnp3-1 ade6-M210 ch16*	This work
MS468	*h^-^*	*leu1-32 cnp3-GFP-leu1^+^*	This work
MS497	*h^-^*	*leu1-32 GFP-cnp1-hph mis16-53*	This work
MS503	*h^-^*	*leu1-32 GFP-cnp1-hph mis18-818*	This work
MS508	*h*	*leu1-32 GFP-cnp1-hph mis6-302*	This work
MS525	*h^-^*	*leu1-32 lys*::*nmtGFP-cnp1-lys1^+^ cnp3-3HA 6his-leu1^+^*	This work
MS527	*h^-^*	*leu1-32 lys*::*nmtGFP-cnp1-lys1^+^ cnp3-1-3HA 6his-leu1^+^*	This work
MS532	*h^-^*	*leu1-32 GFP-cnp1-hph mis6-302 cnp3-1*	This work
MS533	*h^-^*	*leu1-32 GFP-cnp1-hph mis16-53 cnp3-1*	This wor
MS534	*h^-^*	*leu1-32 GFP-cnp1-hph cnp3-1 mis18-818*	This work
MS544	*h^-^*	*leu1-32 cnp3-1-GFP-leu1^+^*	This work
MS545	*h^-^*	*leu1-32 GFP-cnp1-hph cnp3-1mCherry-leu1^+^ mis18-818*	This work
MS546	*h^-^*	*leu1-32 GFP-cnp1-hph cnp3-mCherry-leu1^+^ mis18-818*	This work
MS548	*h^-^*	*leu1-32 GFP-cnp1-hph cnp3-mCherry-leu1^+^*	This work
MS549	*h^-^*	*leu1-32 GFP-cnp1-hph cnp3-1-mCherry-leu1^+^*	This work
MS568	*h^-^*	*leu1-32 Sad1-mCherry<<kanR* transformed with *pREP81-cnp3-GFP*	This work
MS569	*h^-^*	*leu1-32 Sad1-mCherry<<kanR* transformed with *pREP81-cnp3-1-GFP(S508F)*	This work
MS615	*h^-^*	*leu1-32 GFP-cnp1-hph mis12-mCherry-leu1^+^*	This work
MS616	*h^-^*	*leu1-32 GFP-cnp1-hph mis12-mCherry-leu1^+^ cnp3−1*	This work
MS617	*h^+^*	*leu1-32 GFP-cnp1-hph mis12-mCherry-leu1^+^cnp3−1 mis18-818*	This work
MS618 *		*leu1-32 GFP-cnp1-hph mis12-mCherry-leu1^+^ mis18-818*	This work
MS620	*h^-^*	*leu1-32 GFP-cnp1-hph mis12-mCherry-leu1^+^ mis18-262*	This work
MS621		*leu1-32 GFP-cnp1-hph mis12-mCherry-leu1^+^ cnp3−1mis18-262*	This work
TK5	*h^-^*	*leu1-32*::*nmt1GFP-cnp1-leu1^+^*	This work
TK85	*h^-^*	*leu1-32 ade6-M210 ch16*	Laboratory stock

* : The mating type was not determined.

For tagging GFP to the N-terminal of Cnp1, the *cnp1^+^* gene was PCR-amplified with the primers, Cnp1-F and Cnp-R (Table 3), and cloned into pREP41-GFP(N-end tagging) after digestion with restriction enzymes, Sal I and Not I. The resulting plasmid was used to construct MS02 (Table 1), in which GFP-Cnp1 could be conditionally induced. Similarly, the *cnp1^+^* gene was PCR-amplified with the primers, GFP-Cnp1-F and GFP-Cnp-R (Table 3), and cloned into pBR322-(*leu1^+^*) lacking the Bam HI site. The resulting plasmid was integrated at the *leu1* locus to construct TK5 (Table 1).

To construct other plasmids and strains for expression of tagged Cnp1 and Cnp3, similar strategies were employed. The primers and plasmids used for these purposes were summarized in Table 3. All integrants were examined for their integrated locus by PCR as well as crossing.

### Western blotting

Cell extracts were prepared from exponentially growing *S.pombe* cells. The cells were suspended in lysis buffer (25 mM HEPES-KOH at pH 7.5, containing 200mM NaCl, 10% glycerol, 0.2% NP-40 and protease inhibitors (Nacalai Tesque), 1 mM PMSF, and 1% Trasylol) were disrupted by Beads Smash 12 (WAKENYAKU) at 4°. After centrifugation the supernatants were used for SDS-polyacrylamide gel electrophoresis (SDS-PAGE). The extracts were separated by SDS-PAGE gel and blotted to nitrocellulose membranes (Funakoshi). Antibodies for western blotting were diluted as follows: mouse anti-GFP (Roche) 1:1000, anti-HA (12CA5, Roche) 1:3000; and TAT1 anti-α-tubulin (gift from K. Gull, Oxford, UK) 1:5000. Blots were developed using ECL reagents, Substrate Plus (Pierce) and ECL prime (GE Healthcare). The intensity of each band was measured by Image J and indicated arbitrarily.

### Microscopy

*S. pombe* strains were grown in YES or EMM2 medium. Images were acquired on a KEYENCE (BZ-9000) and Delta Vision (Applied Precision LLC, Issaquah, WA, USA), CH350L CCD camera (Photomettric, Tucson, AZ, USA). FISH analyses was performed as described (Funabiki *et al.* 1993).

### Preparation of digoxigenin-labeled probe DNA

pRA140 (Chikashige *et al.* 1989) was used to visualized the centromere. The DNA fragments used to prepare the probes were digested by a mixture of restriction enzymes, AluI, *Dde*I, *Hae*III, *Rsa*I, and Sau3AI to yield an average fragment size of 300bp.These fragment were labeled by digoxigenin-dUTP using the random priming labeling kit(Takara 6045 random primer DNA labeling kit ver.2). Nonreacted nucleotides were removed by microspin G-25 column (GE healthcare).

### Mini-chromosome stability assay

The stability of mini-chromosome was determined by a method developed by (Allshire *et al.* 1995). First, the cells were plated on YE (for Table 2) or EMM containing 10 μg/ml adenine to determine the initial percentage of the cells carrying the mini-chromosome. After incubation in the non-selective medium (Ade^+^), the final percentage of the cells carrying the mini-chromosome was determined. The rate of mini-chromosome loss per division was estimated using the formula: loss rate = 1-(F/I)^1/N^ (F, the final percentage of cells carrying mini-chromosome; I, the initial percentage of cells carrying mini-chromosome; N, the number of generations between F and I).

**Table 2:** Stability of mini-chromosome. The stability of the mini-chromosome Ch16 was determined on YE media. (WT: TK85, *cnp3-1*: MS413)

		Loss rate		
	Background	Per division	Percent division (%)	Fold increase in loss rate
**26°C**	**WT**	**1.4x10^−4^**	**0.014**	**1**
	***cnp3-1***	**3.4x10^−2^**	**3.4**	**243**
**36°C**	**WT**	**2.3x10^−4^**	**0.023**	**1**
	***cnp3-1***	**1.2x10^−1^**	**12**	**522**

**Table 3:** Plasmids and primers used in this study

*1	*pREP1-GFP-Cnp1*
*2	*pREP1-GFP-Cnp1-pUM1*
MS-1	*nmt1-GFP-cnp1*-pBR322
MS-2	*cnp3-mCherry*-pBS
MS-3	*cnp3-1mCherry*-pBS
MS-4	*cnp3-HA*-pBS
MS-5	*cnp3-1HA*-pBS
MS-6	*pREP81-cnp3-GFP*
MS-7	*pREP81-cnp3-1-GFP*

Cnp1-F	ATAGTCGACTATGGCAAAGAAATCTTTAATG
Cnp1-R	ATAGCGGCCGCTCAAGCACCACGAATCC
GFP-Cnp1-F	ATAGGATCCTATGAGTAAAGGAGAAGAAC
GFP-Cnp1-R	ATAGGATCCTCAAGCACCACGAATCCTC
Cnp3-F	ATACTCGAGATGTAGACGATGAATGAAACGTC
Cnp3-R	ATAGCGGCCGCGTCGTTCGTTTGGAAAATC
Rep81Cnp3-F	ATACATATGTTCTAGAGATGAAGATTCCG
Rep81-Cnp3-R	ATAGCGGCCGCGTCGYYCGTTTGGAAAATC

* 1, 2 (Kitagawa *et al.* 2014)

### Chromatin immunoprecipitation (Chip) assay

ChIP was performed as described previously (Kitagawa *et al.* 2014).

### Isolation of the *cnp3-1* mutant

A strain (TK5: *h^-^ leu1-32*::*nmt1-GFP-cnp1^+^* -leu1*^+^*) which was able to express fission yeast CENP-A, Cnp1, tagged with GFP from the nmt1 promoter, was chemically mutagenized as described (Matsumoto and Beach 1991). Exponentially growing cells suspended in TM buffer (50 mM Tris malate (pH 6.0) were treated with NTG (500 µg/ml) at 26° for 20 min and washed three times with TM buffer. Cells were grown in the YES liquid medium for 4h at 26° and washed with EMM2 medium containing thiamine. Cells were grown in the same medium thiamine and grown at 26°. The survivors were plated on the EMM2 plates containing thiamine and grown at 26°. They were subsequently transferred onto EMM2 plates with and without thiamine by replica and grown at 36°. The strains that exhibited temperature sensitivity only when GFP-Cnp1 was expressed were selected. They were then individually examined under a fluorescence microscope. We scored mutants with abnormal distribution of GFP-Cnp1 in the nucleus. Tetrad analysis indicated that five strains including the *cnp3-1*mutant carried a single mutation responsible for the phenotypes (temperature sensitivity and abnormal distribution of GFP-Cnp1).

### Isolation of the *cnp3^+^*gene

By using the temperature sensitivity as a selection marker, a genomic DNA fragment complementing the temperature sensitivity was isolated from a fission yeast genomic DNA library. The integration mapping proved that this fragment originated from the *cnp3-1* locus. A PCR-based strategy was used to identify the mutation site within the *cnp3^+^* coding sequence. The *cnp3-1* mutant gene contained mutation of S508.

### Data availability

All strains and plasmids are available upon request. All data necessary for evaluating our conclusions are represented within this paper. Supplemental material available at Figshare: https://doi.org/10.25387/g3.6618920.

## Results

### Isolation of *cnp3-1*

A strain (TK5: *h^-^ leu1-32*::*nmt1-GFP-cnp1^+^* -leu1*^+^*) which was able to express fission yeast CENP-A, Cnp1, tagged with GFP from the nmt1 promoter was chemically mutagenized and survivors were screened for mutants, which became temperature sensitive upon overexpression of GFP-Cnp1. In this study, we characterized one of the mutants identified through the screen.

**Figure 1:**
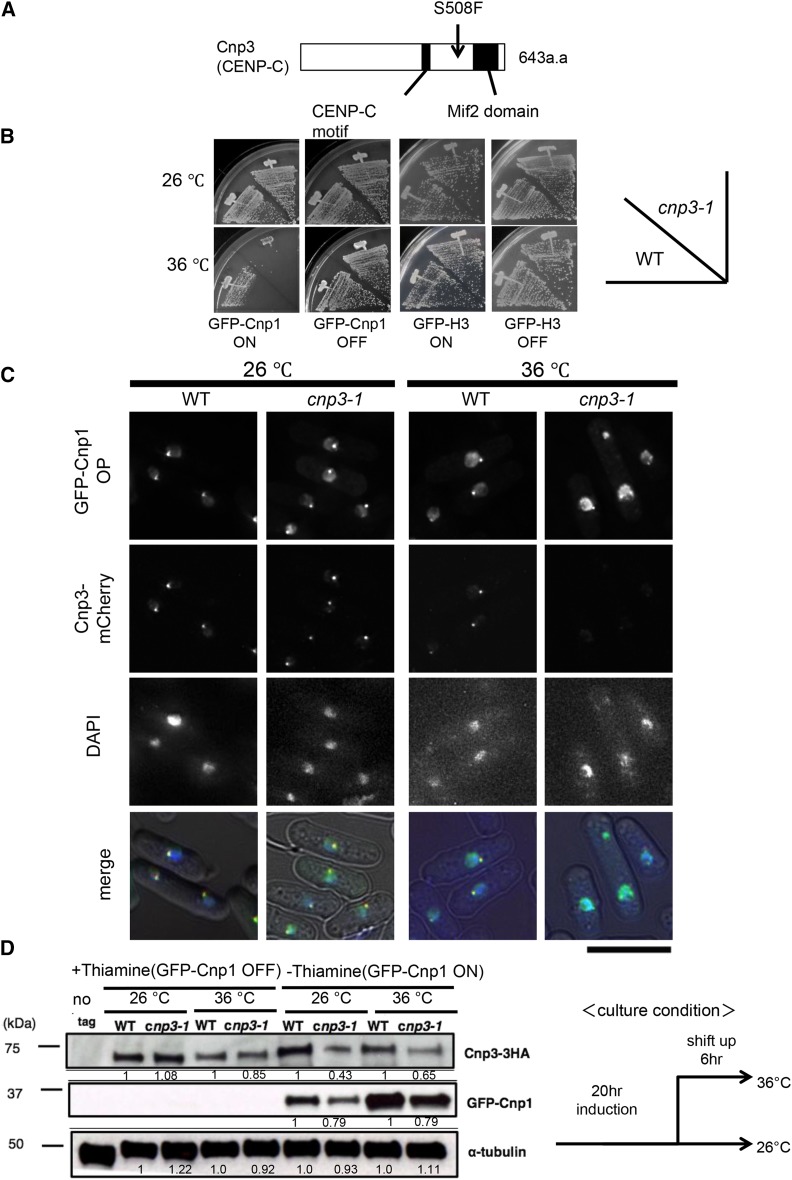
Distribution of GFP-Cnp1. (A) Schematic illustration of the structure of Cnp3 protein and the position of the *cnp3-1* mutation. (B) The wild type strain (WT) and the *cnp3-1* mutant (*cnp3-1*) were transformed with pREP41-GFP- Cnp1 or GFP- H3. The resulting transformants (MS01, MS02, MS03 and MS04) were streaked as indicated and grown at 26°C or 36°C on EMM 2 media with thiamine for repression (GFP-Cnp1 OFF) or without thiamine for derepression (GFP-Cnp1 ON). (C) Localization of GFP-Cnp1 and Cnp3-mCherry were examined in the wild type (MS213) and *cnp3-1* cells (MS217). They were grown at 26°C for 20 hr for induction of GFP-Cnp1 in absence of thiamine, and shifted to 36°C for 6 hr in absence of thiamine. The bar indicates 5 μm. (D) The levels of GFP-Cnp1and Cnp3-HA were examined by western blot in the wild type strain (WT: MS525) and the *cnp3-1* mutant (*cnp3-1*: MS527). They were grown as (C) and the samples were taken 20 hr after the induction at 26 °C and 6 hr after the shift to 36 °C. α-tubulin was used as a loading control.

Genetic analysis and subsequent gene cloning revealed the temperature sensitivity upon overexpression of Cnp1 to be attributable to a mutation on the *cnp3^+^* gene encoding a fission yeast homolog of CENP-C. The mutant is thus designated *cnp3-1* hereafter. Sequencing of the *cnp3-1* mutant gene identified a single point mutation changing TCT of the 508^th^ codon to TTT, resulting in the change in the amino acid from serine to phenylalanine (Figure 1A). As shown in Figure 1B, the *cnp3-1* mutant becomes temperature-sensitive upon overexpression of Cnp1, but not histone H3, demonstrating the specific sensitivity of the *cnp3-1* mutant to Cnp1.

### GFP-Cnp1 foci in the *cnp3-1* mutant

**Figure 2:**
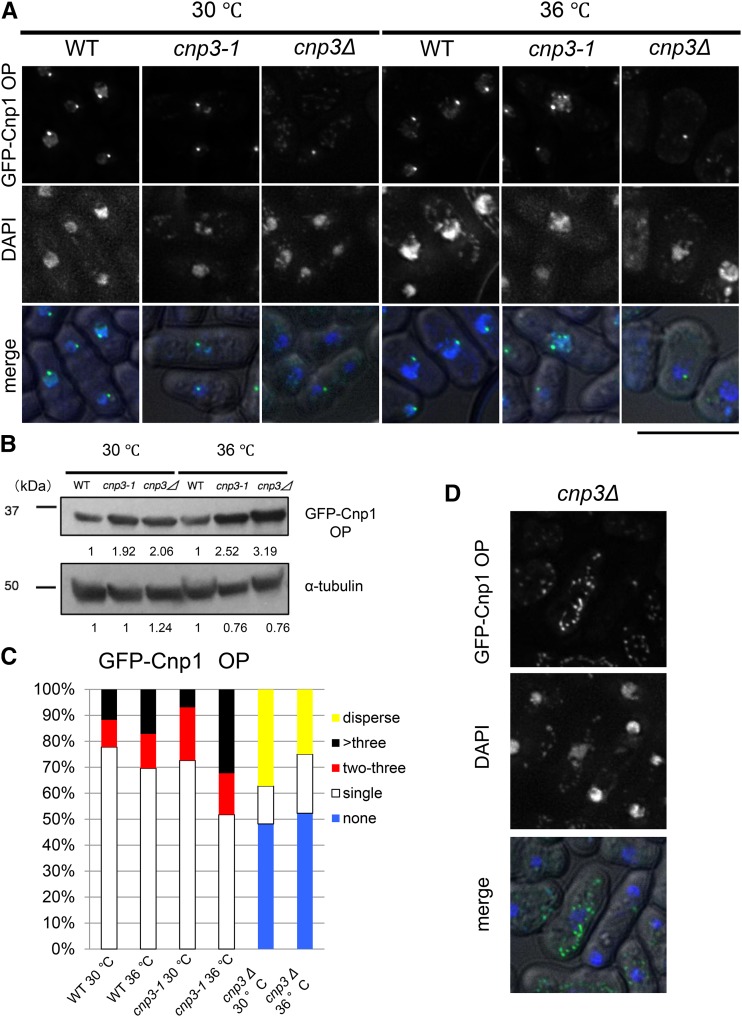
Comparison between *cnp3-1* and *cnp3 Δ*. (A and D) (A) Localization of GFP-Cnp1 were examined in WT (MS216), *cnp3-1* (MS262) and *cnp3Δ* (MS168) cells. They were grown at 30 °C for 20 hr for induction of GFP-Cnp1 in absence of thiamine, and shifted to 36 °C for 6 hr in absence of thiamine. The GFP-Cnp1 appeared as cytoplasmic speckles in the *cnp3Δ* cells (MS168) was shown in (D). The bar indicates 5 μm. (B) The level of GFP-Cnp1 was examined by western-blot. α-tubulin was used as a loading control. The samples were taken 20 hr after the induction at 30 °C and 6 hr after the shift to 36 °C. (C) The statistic analysis of (A).

In order to examine distribution of ectopically expressed GFP-Cnp1 in the nucleus, we observed fission yeast cells overexpressing GFP-Cnp1 microscopically in various genetic background (Figure 1C and Figure 2A). We also examined the level of GFP-Cnp1 by immunoblot with an antibody to GFP. As shown in Figure 1D, GFP-Cnp1 was expressed in the *cnp3-1* mutant at a level similar to that in the wild type strain at 26° and 36°. Likewise, it was expressed at comparable levels in the three strains (wild type, *cnp3-1* mutant and deletion strain for the *cnp3^+^* gene) at 30° and 36° (Figure 2B).

Because three centromeres of fission yeast cluster adjacent to the spindle pole body, SPB (Funabiki *et al.* 1993), the fluorescence signal of GFP-Cnp1 expressed from the native promoter appears as a discrete single speckle in each cell. Microscopic observation revealed that the signal of GFP-Cnp1 appeared as a single speck in most of the wild type cells (78% at 30° and 70% at 36°) even when GFP-Cnp1 was overexpressed (Figure 2A and 2C), suggesting that GFP-Cnp1 was incorporated into the native centromeres, but not into non-centromeric arm regions.

While distribution of the signal from GFP-Cnp1 in the *cnp3-1* mutant at 26 or 30° appeared similarly to that in the wild type cells, it changed dramatically upon the shift to the restrictive temperature, 36° for 6 hr. As shown in Figure 1C, Figure 2A and 2C, the fluorescent signal from GFP appeared as multiple foci (48%), suggesting that the *cnp3-1* mutant is prone to promiscuous accumulation of GFP-Cnp1. We also examined distribution of ectopically expressed GFP-Cnp1 in the deletion strain for the *cnp3^+^* gene (*cnp3Δ*). The *cnp3Δ* strain is viable at 30°, but not at 36°. As shown in Figure 2A and C, no GFP-Cnp1 focus was observed in approximately 50% of the *cnp3Δ* cells. In the remaining 50% of the cells, GFP-Cnp1 appeared as a single speckle or cytoplasmic speckles (Figure 2D). Distribution of GFP-Cnp1 was thus strikingly different between the *cnp3-1* mutant and *cnp3Δ* strain. We therefore speculate that the *cnp3-1* mutation may not be a simple loss of function.

### Characterization of Cnp3-1 mutant protein

**Figure 3:**
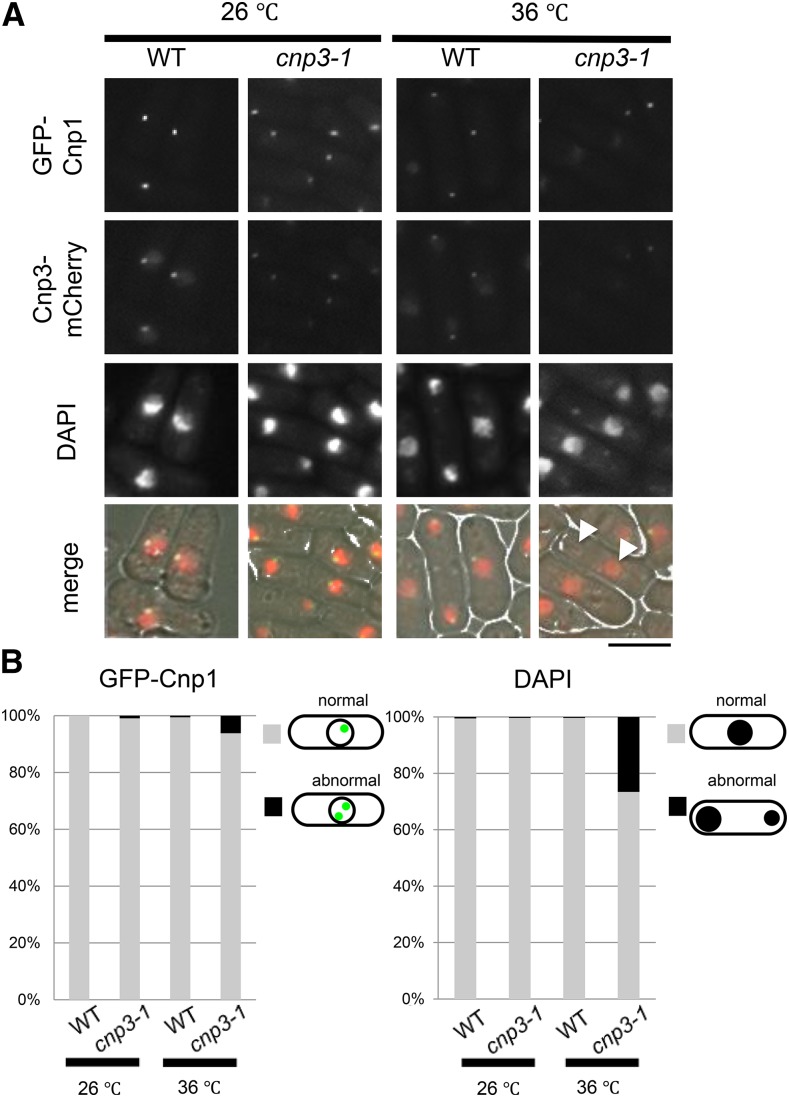
Characterization of the *cnp3-1* mutant. (A) The strains expressing GFP-tagged Cnp1 and mCherry-tagged Cnp3 (WT, MS548), Cnp3-1 mutant protein (*cnp3-1*, MS549) from the native promoter were grown at 26°C or 36°C for 24 hr and observed under a fluorescence microscope. The white arrow heads indicate mis-segregated chromatin mass scored as abnormal in (B). The bar indicates 10μm. (B) The statistic analysis of (A).

We next attempted to investigate the *cnp3-1* mutant under physiological conditions. The experiments described below were performed with cells expressing Cnp1 from the native promoter, unless otherwise stated. As described previously, tagging GFP to the N-terminal of Cnp1 does not affect growth of fission yeast (Takayama *et al.*, 2008). To visualize the Cnp3 protein, mCherry was tagged to the C-terminus of Cnp3 and Cnp3-1 mutant protein, respectively. When Cnp1 is solely expressed from its native promoter, the *cnp3-1* mutant is able to grow at 36°. Microscopic observation of the mutant at 36°, however, revealed that 1) although the signal from GFP-Cnp1 expressed from the native promoter appeared as a single speckle, the intensity of the fluorescent signal was reduced, 2) the level of Cnp3-1 mutant protein at the centromere significantly decreased, and 3) chromosomes segregated unequally in 20% of the cells (Figure 3A and B).

**Figure 4:**
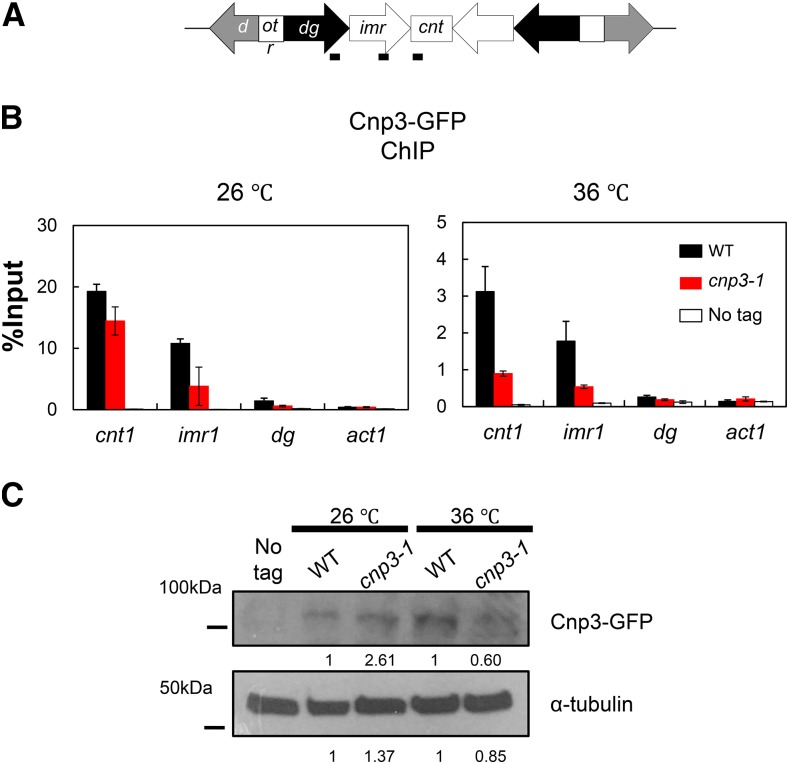
Loss of Cnp3-1 from the centromere. (A) Schematic illustration of the centromere I (Cen I). Each centromere contains a central domain (cnt), flanked by innermost repeats (imr). This core domain is surrounded by arrays of outer repeats (otr). The black bars indicate the position of the primers used in the ChIP analysis. (B) Localization of Cnp3-GFP in Cen I was examined by ChIP analysis in the wild type stain (WT: MS468) and the *cnp3-1* mutant (*cnp3-1*: MS544). The stains were grown at 26 °C and shifted to 36 °C for 6 hr. (C) The level of Cnp3-GFP was examined by western-blot in the wild type strain (WT: MS468) and the *cnp3-1* mutant (*cnp3-1*: MS544) grown as in (B). α-tubulin was used as a loading control.

**Figure 5:**
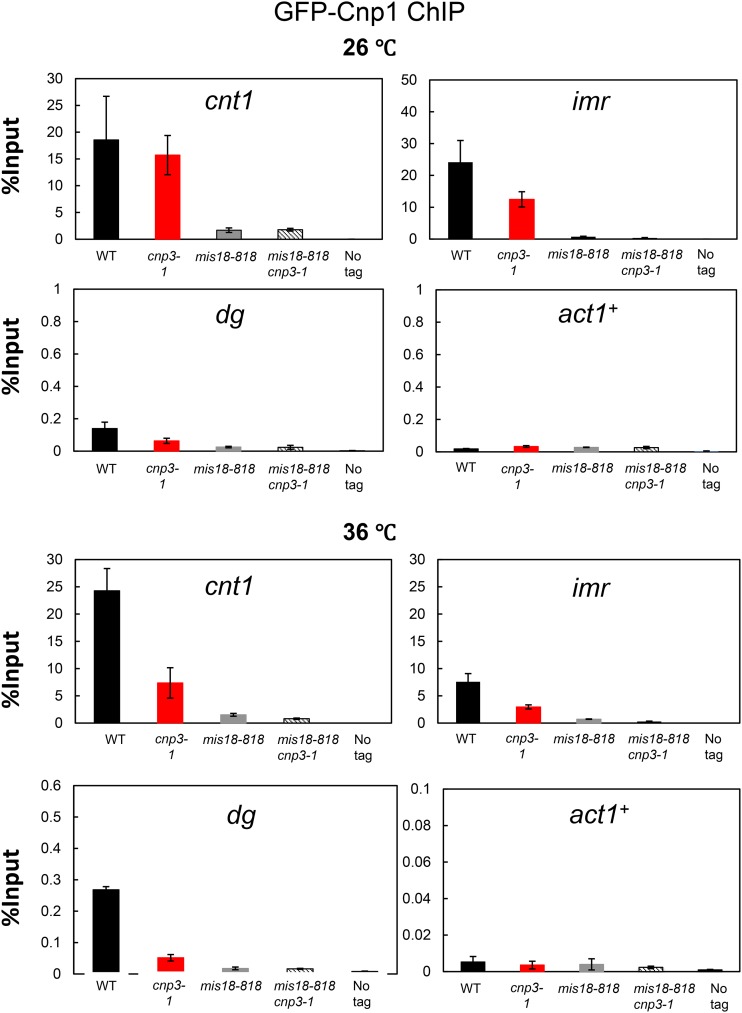
Distribution of Cnp1 at the centromere. Distribution of GFP-Cnp1 at *Cen I* was examined by ChIP analysis in the indicated mutants (WT: MS343, *cnp3-1*: MS344, *mis18-818*; MS503 and *cnp3-1 mis18-818*: MS534) grown as in Fig. 4B.

GFP was tagged to Cnp3 and Cnp3-1, respectively, and examined by immunoblot as well as ChIP (Figure 4A and C). As shown in Figure 4B and C, the level of Cnp3-1 mutant protein slightly reduced at 36°. ChIP analysis indicated that the amount of Cnp3-1 bound to centromere chromatin decreased upon the shift to the restrictive temperature (Figure 4A and B). ChIP analysis for Cnp1 also indicated that the amount of Cnp1 bound to centromere chromatin significantly decreased in the *cnp3-1* mutant at 36° (Figure 5).

**Figure 6:**
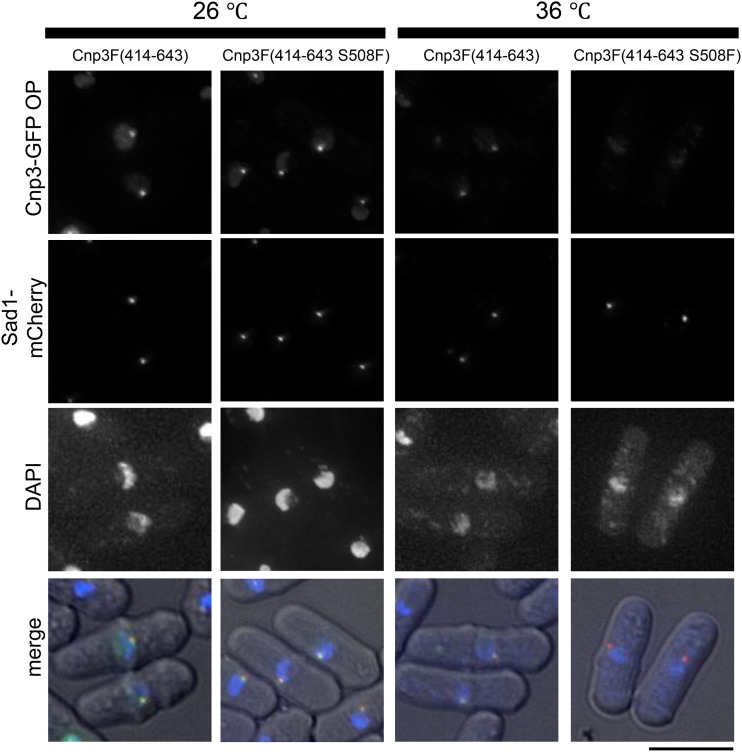
S508 of Cnp3 is important for centromere-targeting. Localization of a partial fragment of Cnp3 spanning from 414 to 643 amino acids tagged with GFP were examined at 26°C or 36°C in the wild type strain (MS568 and MS569). Sad1 tagged with mCherry was used as a marker for the centromere cluster. These strain were grown as in Fig. 1C. The bar indicates 5 μm.

The centromere binding activity was mapped at a domain of Cnp3 spanning from 414 to 643 amino acids (Tanaka *et al.* 2009). This domain was fused with GFP, designated F(414-643)-GFP, and expressed in the wild type strain. As shown in Figure 6, F(414-643)-GFP was colocalized with SPB, a marker for the cluster of the three centromeres. When the mutation of *cnp3-1* (S508F) was introduced in the domain, designated F(414-643 S508F)-GFP, it was colocallized with SPB at 26°, but not at 36°, indicating that F(414-643S508F)-GFP could recapture the phenotype of the full length Cnp3-1 mutant protein.

These results suggested that the Cnp3-1 mutant protein lost its binding activity to the centromere and allowed a partial loss of Cnp1 from the centromere at 36°, and prompted us to examine the function of the centromere in the mutant. As shown in Table 2, the stability of the mini-chromosome Ch16 was reduced in the *cnp3-1* mutant, indicating a low activity of the centromere in the mutant.

### Ectopic foci of Cnp1 in *cnp3-1*

**Figure 7:**
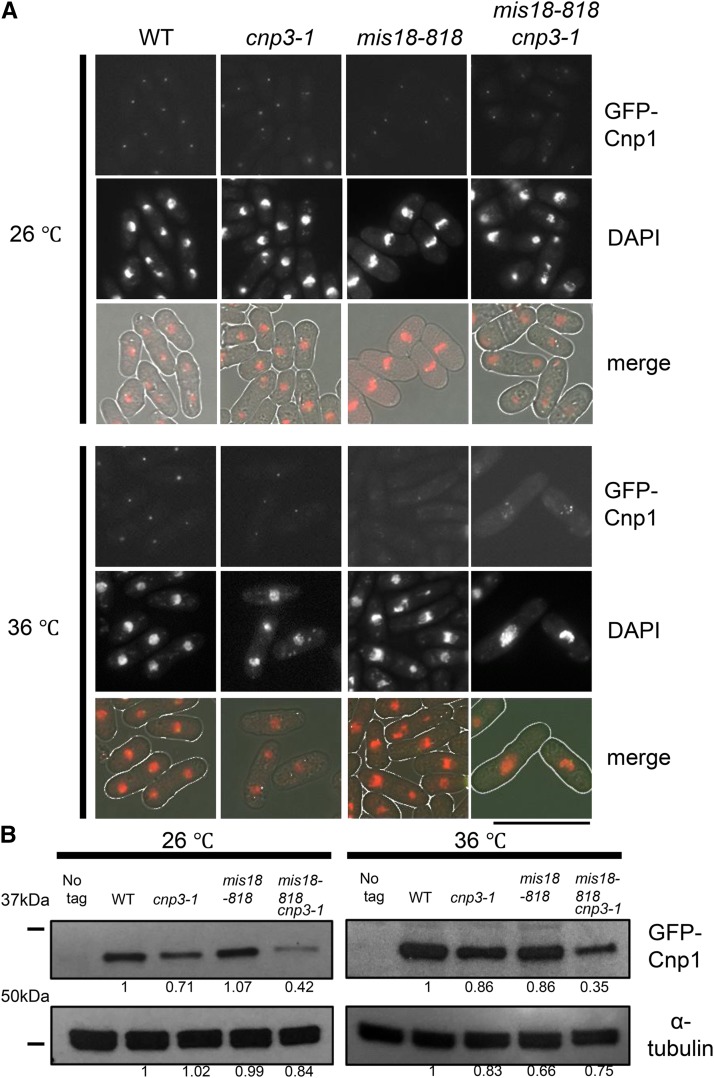
Multiple foci of GFP-Cnp1 in *mis18 cnp3-1*. (A) Cnp1 tagged with GFP was expressed from the native promoter. Each strain (WT: MS343, *cnp3-1*: MS344, *mis18-818*: MS503 and *cnp3-1 mis18-818*: MS534) was grown at 26 °C and shifted to 36 °C for 6 hr. The bar indicates 10 μm. (B) The level of Cnp1 in strains used in (A) was examined by immunoblot. α-tubulin was used as a loading control.

As described above, the *cnp3-1* mutant was prone to promiscuous accumulation of Cnp1 at 36° when Cnp1 was overexpressed. We speculated that when Cnp1 was present in excess, Cnp3-1 mutant proteins might interact with Cnp1 and promote promiscuous accumulation of Cnp1. In order to test this scenario under a physiological condition, we attempted to increase the level of free Cnp1 by disabling a system to incorporate Cnp1 in the centromere chromatin. It has been shown previously that Mis18 is responsible for incorporation of Cnp1 into centromeric chromatin (Hayashi *et al.* 2004; Williams *et al.* 2009). As shown in Figure 7A, the intensity of GFP-Cnp1 foci decreased at 36° in the temperature sensitive *mis18-818* mutant. Remarkably, in the double mutant (*mis18-818 cnp3-1*), multiple foci of GFP-Cnp1, which were brighter than those in the single *mis18-818* mutant, were observed (Figure 7). We speculated that foci of GFP-Cnp1 were formed ectopically in the *cnp3-1 mis18-818* double mutant for the following three reasons; 1) ChIP analysis (Figure 5) indicated that the level of Cnp1 bound to the native centromere was extremely low, 2) the foci were formed even in the *mis18-818* mutant defective in CENP-A deposition in the native centromere, and 3) the number of the foci in a nuclei occasionally exceeded three, the number of the fission yeast centromeres (Figure 7A).

We next examined distribution of Mis12, a protein localized at centromeres in a Cnp1-independent manner (Goshima 2003). As shown in Figure S1, S2 and S3. Mis12 was observed as a discrete single speckle in the *cnp3-1 mis18-818 and cnp3-1 mid18-262* double mutant at 26° (Figure S1) and 36° (Figure S2 and S3), suggesting that the centromere cluster was normally maintained. FISH analysis with a probe for the centromere also indicated that the centromere cluster was normally maintained (Figure S4). Furthermore, multiple foci of GFP-Cnp1 formed at 36° were found at positions different from the speckles of Mis12 (Figure S2), demonstrating that the foci of GFP-Cnp1 in the *cnp3-1 mis18-818* double mutant were ectopic.

As shown in Figure S5 and S6, multiple foci were also observed when the *cnp3-1* mutation was combined with *mis6-302* or *mis16-53*, a mutation defective in deposition of Cnp1 into cenromeric chromatin.

**Figure 8:**
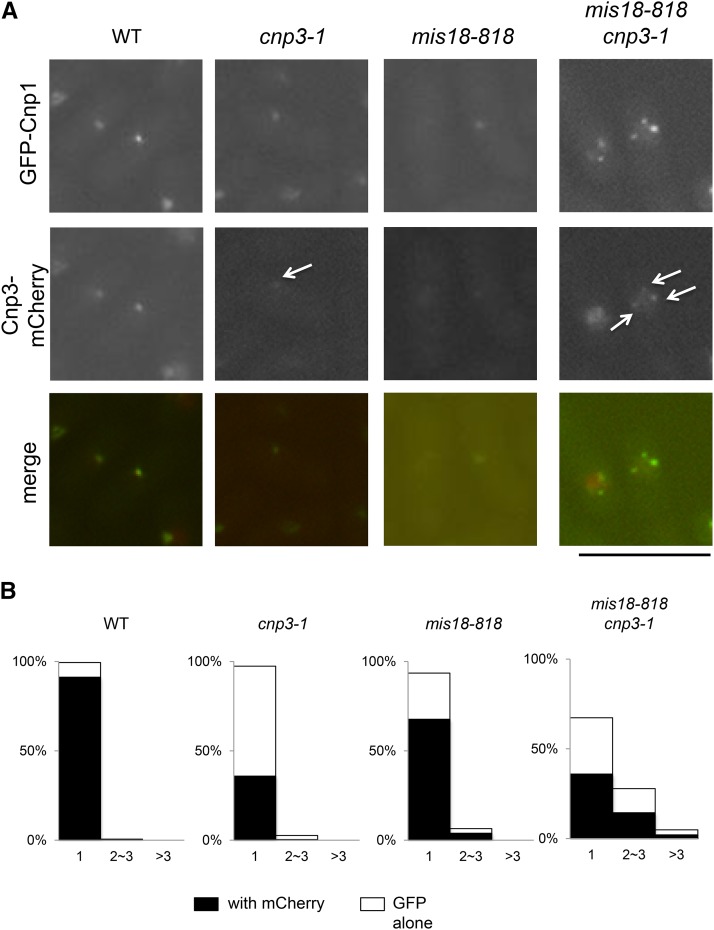
Colocalization of Cnp1-foci and Cnp3-1 in *mis18 cnp3-1*. (A) Both Cnp1 tagged with GFP and Cnp3 (or Cnp3-1) tagged with mCherry were expressed from their native promoters in the four strains (WT: MS548, *cnp3-1*: MS549, *mis18-818*: MS546 and *cnp3-1 mis18-818*: MS545). They were grown as in Fig. 7A. The white arrows indicate the Cnp3 (Cnp3-1)-foci. The bar indicates 10 μm. (B) The statistic analysis of (A).

Finally, we examined direct contribution of Cnp3-1 mutant protein to ectopic formation of Cnp1 foci by localizing Cnp3-1 proteins. In the *cnp3-1* mutant, weak fluorescent signal from Cnp3-1-mCherry was colocalized with GFP-Cnp1, which presumably represented the native centromere, indicating that the Cnp3-1 mutant protein lost the activity to bind to the native centromere (Figure 8A). In the double mutant (*mis18-818 cnp3-1*), the signal from Cnp3-1-mCherry, which was easily discernable, was colocalized with multiple GFP-Cnp1 foci, which were likely ectopic. As shown in Figure 8B, more than 33% of the double mutant cells exhibited multiple foci of GFP-Cnp1.

Based on the observation, we speculated that Cnp3-1 mutant protein preferentially recognized ectopically accumulating GFP-Cnp1 and might promote further accumulation.

## Discussion

In this study we have characterized the mutant of *cnp3^+^* gene (*cnp3-1*), which encodes a fission yeast homolog of mammalian CENP-C, with and without overexpression of Cnp1. The mutation site was mapped at S508, which is within the DNA binding domain of Cnp3 (Tanaka *et al.* 2009).

When Cnp1 is not overexpressed, the *cnp3-1* mutant is viable from 26 to 36°. Both microscopic observation and ChIP analysis have revealed that the Cnp3-1 mutant protein looses the binding activity to the native centromeres at 36°. In addition, the level of Cnp1 bound to centromeres decreased. It has been shown by an *in vitro* study that human CENP-C stabilizes CENP-A nucleosomes on chromatin through physical interaction (Falk *et al.* 2015). We thus speculate that the reduction in the level of Cnp1 on centromeres is a direct consequence of a loss of the Cnp3-1 mutant protein from centromeric chromatin.

When Cnp1 is overexpressed, the *cnp3-1* mutant is unable to grow at 36°. Microscopic observation revealed that multiple foci of GFP-Cnp1 were assembled in the mutant. Because we found cells (32%) with more than three foci of GFP-Cnp1(Figure 2C), some of the multiple foci, at least, are ectopically assembled. In a vast majority of the *cnp3Δ* strain overexpressing Cnp1, no focus of GFP-Cnp1 was observed, indicating that loss of function of the *cnp3^+^* gene does not result in promiscuous accumulation of Cnp1. We therefore speculate that the single amino acid change, S508F, in Cnp3 causes a gain of function mutation.

The Mis18 protein forms a complex with Mis16 and is required for centromere localization of Scm3, a Cnp1-chaperone(Hayashi *et al.* 2004; Williams *et al.* 2009). Consistently with the function of Mis18, incorporation of GFP-Cnp1into centromeric chromatin was significantly reduced in a temperature sensitive mutant, *mis18-818*. Interestingly, when the *mis18-818* mutation was combined with the *cnp3-1* mutation, which is also defective in incorporation of Cnp1 into the native centromeres, multiple Cnp1-foci were observed. As Mis12, a protein localized at the centromere independently from Cnp1, appeared as a single speck, the cluster of the native centromeres is normally maintained in the double mutant, *mis18 cnp3-1*. Multiple Cnp1-foci therefore are ectopic. Furthermore, the Cnp3-1 mutant protein is associated with these foci, suggesting that it may contribute to ectopic accumulation of Cnp1.

It has been demonstrated that human CENP-A is overexpressed in some aggressive cancer cells, where it can be promiscuously incorporated in non-centromeric regions in the form of heterotypic nucleosomes containing H3.3 (Lacoste *et al.* 2014). The heterotypic nucleosomes is as stable as the CENP-A homo-nucleosome and can bind to CENP-C *in vitro* (Arimura *et al.* 2014). Furthermore, Cnp1 accumulation normally depends on Cnp3 (Tanaka *et al.*, 2009). Taking these reports and our results together into consideration, we speculate that 1) the Cnp3-1 mutant has lost the specific binding activity to the native centromere, where the CENP-A homo-nucleosome is a major component in chromatin and 2) it binds to the heterotypic nucleosomes containing H3.3 more efficiently than the wild type Cnp3 protein, and finally 3) when Cnp1 is present in excess (in cells overexpressing Cnp1, or in the double mutant, *mis18 cnp3-1*, where deposition of Cnp1 into the native centromeres is prevented), the Cnp3-1 protein preferentially binds to and stabilizes the heterotypic nucleosomes promiscuously accumulated on non-centromeric regions. Alternatively it is possible that the Cnp3-1 protein may stabilize the CENP-A homo-nucleosomes promiscuously deposited at non-cetromeric regions.

With the specific binding activity to the native centromere, Cnp3 serves as a part of the epigenetic mechanism to restrict the position of CENP-A accumulation and prevents formation of *de novo* centromeres.
